# Detecting “invisible” *Phytophthora* lineages in publicly available sequencing data

**DOI:** 10.1093/ismeco/ycag019

**Published:** 2026-01-30

**Authors:** Tage Rosenqvist, Michelle Cleary

**Affiliations:** Southern Swedish Forest Research Centre, Swedish University of Agricultural Sciences, P.O. Box 190, Alnarp, Lomma SE-234 22, Sweden; Southern Swedish Forest Research Centre, Swedish University of Agricultural Sciences, P.O. Box 190, Alnarp, Lomma SE-234 22, Sweden

**Keywords:** *Phytophthora*, internal transcribed spacer, metagenomics, eukaryotic diversity, monitoring

## Abstract

Our understanding of microbial eukaryotic diversity is limited by biases induced by cultivation and DNA-amplification. Microbial lineages which are challenging or impossible to culture and develop universal metabarcoding primers for can be considered “invisible.” These “invisible” microbes can however be detected in genomic and metagenomic sequencing datasets. This study introduces a new pipeline for targeted assembly of internal transcribed spacer (ITS) sequences from genomes and metagenomes (https://github.com/tage-ro/denim), which provides advantages in sensitivity and precision over comparable marker-gene assembly software. It further shows how publicly sequencing datasets can be screened for the genus *Phytophthora*, which includes economically and ecologically devastating plant pathogens. Analysis of 104 sequencing datasets resulted in 733 full ITS sequences, 1626 ITS1 sequences and 2191 ITS2 sequences associated with a variety of eukaryotic lineages. *Phytophthora* ITS sequences associated with known species in clades 1, 2, 4, 6, 7 and 8 were assembled, along with sequences only distantly related to known taxa. In addition, it provided potential indications of new pathogen-host interactions, with potential impacts on agriculture and human health. This study presents a new approach towards discovering and detecting “invisible” microbes, thus expanding our understanding of microbial eukaryotic diversity. Moreover, it allows detection and monitoring of new host–microbe interactions, and characterizing the geographic distribution of cultured and uncultured microorganisms.

## Graphical Abstract

**Graphical Abstract:**
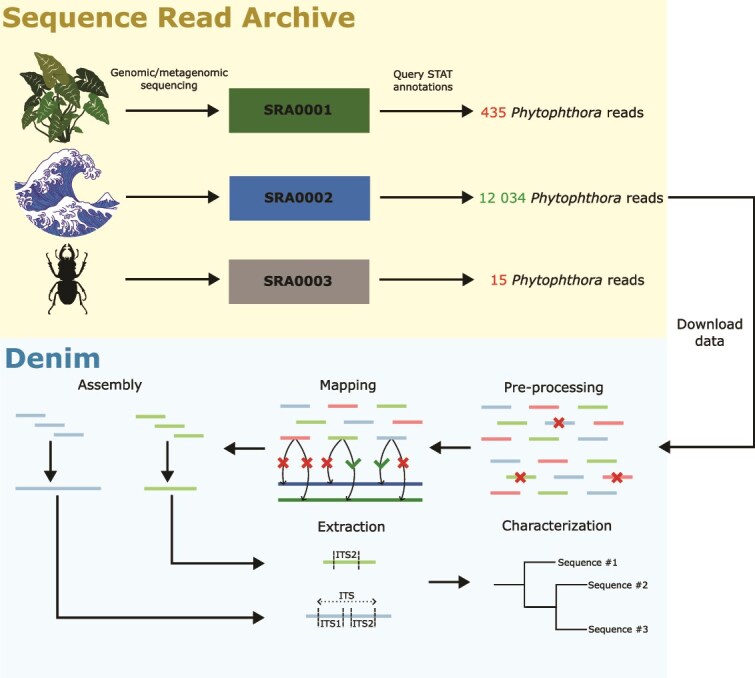

## Introduction

Stramenopiles of the phylum *Oomycota* include some of the world’s most economically and ecologically devastating pathogens of plants and animals, impacting agriculture [1], aquaculture [2] and forestry [3], with *Pythium insidiosum* being capable of causing life-threatening infections in humans and other mammals [4]. Members of genus *Phytophthora* are particularly potent threats to a variety of herbaceous and woody plants in natural and agricultural environments [5].

All methods of detecting and analysing *Oomycota* are biased. Conventional microbial culturing favours fast-growing microbes which are tolerant of the conditions present in the culturing media and does not permit the study of obligate biotrophs, a common lifestyle within *Oomycota* [6]. This causes culturing bias, which severely affects studies of eukaryotic communities in marine environments [7] and in the human gut [8]. Studies of *Oomycota* often enrich their abundance through baiting prior to culturing, representing an additional source of bias [9]. In contrast, metabarcoding approaches allow the study of *Oomycota* in their natural environments by amplifying and sequencing specific genetic markers, such as the internal transcribed spacer (ITS) regions of rRNA genes. However, the choice of primers used for PCR amplification greatly influences the outcome of these studies, as different lineages of *Oomycota* are amplified with differing efficiency [10]. Primer bias is in turn implicitly linked to culturing bias, as PCR primers for metabarcoding have often been developed and validated using cultured isolates [11]. Microbes which are neither culturable nor amplified by established primer sets will thus never be observed by either method. These “invisible” microbes represent a potentially significant blind spot in global efforts to limit and monitor the spread of pathogenic microbes.

Several factors suggest “invisible” microbes could exist within the phylum *Oomycota*. Obligate biotrophy, a common feature in the phylum, prohibits observations through cultivation-based analysis. Cultivation-independent methods of identification, such as metabarcoding, are less developed than for other groups, such as *Fungi* [12], and are complicated by length variations in the sequences of key genomic markers [13]. In addition, while many species within *Oomycota* produce visible structures which permit detection and identification without cultivation or DNA analysis, pathogens with less conspicuous lifestyles may go undetected. Examples include *Phytophthora cinnamomi*, a soil-borne species which can infect plants without causing symptoms [14]. As species in *Oomycota* can be unculturable, difficult to detect using culture-independent methods, and can lack visible structures, a lineage with these combined characteristics would be “invisible” to many methods of analysis.

The challenges posed by culturing and PCR biases are not unique to *Oomycota*. In studies of prokaryotes, they have been overcome through shotgun metagenomics, which allows near-complete genomes to be assembled from non-amplified environmental sequencing data [15]. While metagenome assembled genomes (MAGs) have also been generated for eukaryotes [16], the larger genome sizes and (generally) lower abundances of eukaryotes makes assembly of complete genomes from metagenomic data difficult. When eukaryotic MAGs are generated, establishing their taxonomic identity is complicated by the reliance of established taxonomies on phylogenetic relationships between genetic markers such as ITS, which may not be recovered in MAGs.

Assembling marker genes from metagenomic data allows the detection and identification of “invisible” microbes. Several software pipelines have been developed for this purpose: EMIRGE [17], MATAM [18], MetaRib [19] and phyloFlash [20] reconstruct small subunit (SSU) rRNA gene sequences by mapping reads from shotgun sequencing to taxonomically annotated reference databases and assembling the mapped reads. While comprehensive, well-annotated databases of SSU rRNA genes exist for prokaryotes and eukaryotes [21], the high degree of conservation within some groups, such as the kingdom *Fungi*, limits the utility of the SSU rRNA gene as a taxonomic and phylogenetic marker [22]. It would thus be desirable to assemble more informative markers, such as ITS sequences.

In this study, we developed a pipeline for de-novo-assembly of ITS sequences from metagenomic data, which we named denim. We benchmarked the ability of denim to produce informative marker sequences from a synthetic metagenome composed of 10 *Phytophthora* species and compared its’ performance to EMIRGE and phyloFlash. We then proceeded to use denim to assemble ITS sequences from publicly available shotgun sequencing datasets (genomes and metagenomes), in search of “invisible” *Phytophthora* lineages. By making denim freely available to researchers, we provide a way for the scientific community to identify blind spots caused by culturing- and PCR-bias, increasing our understanding of eukaryotic biodiversity and our preparedness toward emerging plant pathogens.

## Materials and methods

### The denim pipeline

**Figure 1:**
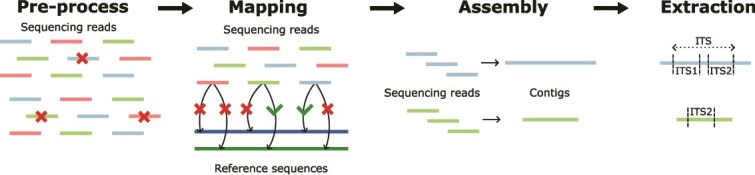
Overview of the denim pipeline. Short sequencing reads are pre-processed through removal of low quality and duplicated reads. Pre-processed reads are then mapped to a reference database. Reads which have 50% or more identity to entries in the reference database are retained and assembled into contigs. Finally, ITS regions are identified and extracted from the contigs.

Executables for the denim pipeline, along with instructions for software installation and usage, are available on GitHub: https://github.com/tage-ro/denim. The denim pipeline follows a similar approach to that of phyloFlash, and consists of four steps: pre-processing, mapping, assembly and ITS sequence extraction (Fig. 1).

Pre-processing, including adapter removal, trimming of low-quality bases, removal of poly-X bases and deduplication is performed by fastp v0.24.0 [23]. For performance purposes, the deduplication calculation accuracy is set to 1.

Mapping of pre-processed reads to the reference database is performed by bbmap v39.06 [24] in “fast” mode. By default, read pairs with at least 50% identity to the reference database are retained. This threshold is lower than that used by phyloFlash (70%) to accommodate higher sequence variability in ITS sequences than SSU rRNA genes, and to allow the recovery of ITS sequences from lineages without close relatives in the used reference database. The reference database used was v10.0 of the UNITE database [25], featuring 156 820 ITS sequences from all eukaryotes. While dominated by the kingdoms *Fungi* (102 137 sequences annotated as *Fungi*) and *Viridiplantae* (35 585 sequences annotated as *Viridiplantae*), 830 sequences originate from the phylum *Oomycota*, out of which 138 sequences are annotated as belonging to the genus *Phytophthora*. As assembly of rRNA regions adjacent to the ITS may aid in subsequent ITS sequence extraction, the “dev” version, which retains these regions, was used.

Reads mapping to the reference database are assembled using MEGAHIT v1.2.9 [26], with k_step set to 4 to improve assembly of low-coverage sequences.

ITS sequence extraction was performed using ITSx v1.1.3 [27]. ITSx occasionally identifies ITS sequences at the edges of contigs, which can be partial or include other regions of rRNA. As such, these sequences were removed.

#### Benchmarking denim against SSU assembly software

The ability of the denim pipeline to assemble phylogenetically and taxonomically useful marker genes from metagenomic data was compared to two pipelines for SSU rRNA assembly: phyloFlash and EMIRGE.

The benchmarking dataset consisted of short reads (2x90 bp) generated from a multi-isolate *Phytophthora* genome sequencing effort (BioProject PRJNA746351) [28]. 10 million reads each were taken from unassembled sequencing data of 10 species of *Phytophthora* (*P. brassicae*, *P. foliorum, P. hibernalis*, *P. melonis*, *P. niederhauserii*, *P. parvispora*, *P. pini*, *P. pisi*, *P. pistaciae* and *P. syringae*) and concatenated into a single FASTQ-file to produce a synthetic metagenome. A list of the utilized genome sequencing datasets is available as Supplementary Table 1. We note that the genomes utilized to benchmark the pipelines are not generated from the ex-types of the species, and may thus not be identical to sequences from the type species.

The dataset generation procedure was motivated by concerns that common methods of generating benchmarking metagenomes, by simulating reads from sets of genome assemblies [20], may create datasets with unrealistically low fractions of reads from rRNA genes as an effect of the repetitive nature of rRNA gene-coding regions, which in eukaryotes can occur in more than 300 000 copies [29]. Their true lengths are not always reflected in genome assemblies as they cannot be directly resolved with short-read sequencing approaches. By creating the benchmarking data from real, unassembled reads, this problem is avoided.

Both phyloFlash and EMIRGE were run within phyloFlash v3.4.2, by executing phyloFlash with the “-emirge” flag. Pipeline performances were assessed by using megablast to query assembled sequences against the NCBI core_nt database on 2026-01-14. The pipelines were compared in their ability to assemble *Phytophthora* marker genes (the number of sequences with at least 95% identity to *Phytophthora* ITS, 18S or mitochondrial 16S sequences) and the utility of the assembled genes to identify the species present in the benchmarking dataset (the number of sequences where the result with the lowest E-value originated from a species which was present in the benchmarking dataset). This method of taxonomic annotation was chosen to allow comparison between different genetic markers. More accurate taxonomic identification could be performed by comparing generated sequences to well-validated databases of marker genes from ex-types and ex-epitypes; however, while this type of database is available for *Phytophthora* ITS sequences [5] it has not been produced for 18S or mitochondrial 16S sequences.

### *Phytophthora* ITS sequences from genomes and metagenomes

To identify sequencing datasets likely to contain novel *Phytophthora* lineages, we used Google BigQuery to search for Sequence Read Archive (SRA) runs containing at least 5000 reads estimated as belonging to *Phytophthora*, as per their Sequence Taxonomic Analysis Tool (STAT) annotations [30]. The following query was used:

SELECT m.acc, m.sample_acc, m.biosample, m.sra_study, m.bioproject, m.library_name, m.librarysource, m.librarylayout, m.libraryselection, m.organism, m.mbases, tax.total_count.

FROM ‘nih-sra-datastore.sra.metadata’ as m, ‘nih-sra-datastore.sra_tax_analysis_tool.tax_analysis’ as tax.

WHERE m.acc = tax.acc AND tax_id = 4783 AND total_count >5000.

When executed (2025-05-30), this query yielded 13 303 SRA accession identifiers. As analysis of all identified datasets would be intractable given the current computational efficiency of denim, we opted to study two subsets of samples:

1. Paired-end non-*Oomycota* genome sequencing datasets containing at least 10 000 reads estimated to belong to *Phytophthora*, representing simple metagenomes, where *Phytophthora* was an unintended contaminant. Analysis of these samples may identify new hosts or obligately biotrophic lineages.
2. Paired-end metagenome sequencing datasets containing at least 10 000 reads estimated to belong to *Phytophthora*, representing environmental samples with significant presence of *Phytophthora*.

One sample was analysed per BioProject. When several samples in a BioProject had over 10 000 *Phytophthora* reads, the sample with the highest ratio of *Phytophthora* reads to total sequenced base pairs was chosen. The selected sequencing datasets were downloaded using the SRA toolkit v3.0.7 [31]. Datasets which could not be split into files containing forward and reverse reads were excluded, resulting in 80 genomic and 24 metagenomic sequencing datasets. A list of the analysed datasets is available as Supplementary Table 2.

These datasets are unlikely to represent all samples in the SRA with abundant *Phytophthora* populations, as the taxonomic coverage of STAT is limited to species with genomes present in the NCBI RefSeq database, which currently (2025-11-06) applies to only five *Phytophthora* species (*P. cinnamomi*, *P. infestans*, *P. nicotianae*, *P. ramorum* and *P. sojae*). As such, only reads matching the genomes of any of these species will be considered as originating from *Phytophthora*, limiting the ability of this approach to identify datasets with abundant *Phytophthora* populations from other lineages.

Sequences identified by ITSx as *Oomycota* were taxonomically annotated against the UNITE database v9.0 using the GBIF sequence ID web tool [32]. To further investigate the phylogeny of assembled ITS sequences, they were aligned to *Oomycota* ITS sequences from the UNITE database v10.0 using MAFFT v7.526 [33]. The generated multiple sequence alignment was then trimmed using ClipKIT v2.2.4 [34] and used as input for phylogenetic tree construction with IQTREE2 v2.4.0-cpeGNU-24.11 [35] with 1000 ultrafast bootstraps (UFboot). This was performed for the full ITS sequences, ITS1 and ITS2 regions separately, resulting in three phylogenetic trees.

Chimeric ITS sequences, i.e. composites of ITS regions from different organisms, can occur as a consequence of sequences shared across lineages, particularly in the highly conserved ~160 bp 5.8S ribosomal rRNA region [36]. Potentially chimeric sequences were identified using uchime2_ref of the USEARCH package v11.0.667 [37] in “high confidence” mode, with the UNITE database (v10.0, non-dev version) as the reference database, as the UCHIME-optimized version of the UNITE database is currently only available for fungal sequences.

### Data analysis

**Figure 2:**
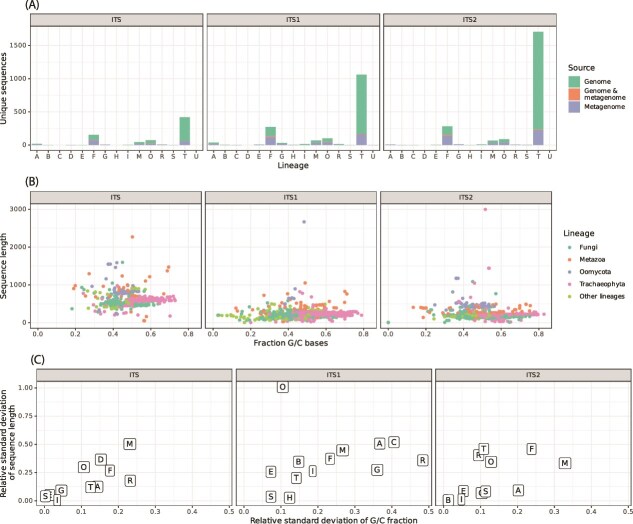
Features of assembled ITS sequences. One-letter codes are used for lineages: A = *Alveolata*, B = *Bryophyta*, C = *Bacillariophyta*, D = *Amoebozoa*, E = *Euglenozoa*, F = *fungi*, G = *Chlorophyta*, H = *Rhodophyta*, I = *Phaeophyceae*, M = *Metazoa*, O = *Oomycota*, R = *Rhizaria*, S = *Synurophyceae*, T = *Trachaeophyta*, U = *Eustigmatophyceae*. (A) Number of unique sequences per lineage, colored by the type of dataset (genomic, metagenomic) from which they were generated. (B) Sequence lengths and G/C contents of sequences, colored by lineage (for the four most common lineages). (C) Variability in sequence lengths and GC contents, as relative standard deviations (standard deviations divided by the means), for all lineages and sequence types.

Downstream data analysis was performed in R v4.4.3 [38]. Figures were created using Inkscape v1.4 [39] (Fig. 1 and the graphical abstract) and ggplot2 v3.5.2 [40] (Fig. 2).

## Results and discussion

### Performance of marker gene assembly pipelines

The benchmarked marker gene assembly pipelines (denim, phyloFlash, EMIRGE) varied in their ability to produce informative gene sequences from the benchmarking dataset containing reads from 10 *Phytophthora* species (Supplementary Table 3).

Denim assembled seven ITS1 sequences and five ITS2 sequences, as well as one full ITS sequence, with at least 95% identity to *Phytophthora* ITS sequences. For six out of the seven ITS1 sequences, the most similar sequence in the core_nt database originated from one out of the 10 species present in the benchmarking dataset (*P. brassicae*, *P. foliorum*, *P. melonis*, *P. pini*, *P. pistaciae*, *P. syringae*). One ITS1 sequence most closely matched *P. sojae*, which was not present in the benchmarking dataset, but is closely related to *P. pisi*. It is possible that the strain annotated as *P. pisi* in the benchmarking dataset (Ppisi_OSU-2014) represents a lineage which is phylogenetically positioned between *P. pisi* and *P. sojae*. All five ITS2 sequences most closely matched sequences originating from species within the benchmarking dataset (*P. brassicae*, *P. parvispora*, *P. pini*, *P. pistaciae*, *P. syringae*), although one sequence matched both *P. pini* and *P. citricola* with 100% identity, highlighting the difficulty of discerning between these species by their ITS2 sequences. No denim sequences matched *P. niederhauserii* or *P. hibernalis*.

EMIRGE generated three sequences with at least 95% identity to *Phytophthora* 18S sequences and three sequences with at least 95% identity to *Phytophthora* mitochondrial 16S sequences. No EMIRGE sequences had sequences originating from species within the benchmarking dataset as their closest match, instead matching *P. cinnamomi*, *P. citricola, P. infestans, P. megasperma* and *P. sojae*. PhyloFlash assembled one sequence with 99.6% identity to *P. sojae* mitochondrial 16S.

In addition to *Phytophthora* marker genes, the pipelines detected potential fungal contaminants within the benchmarking dataset. Denim assembled two full ITS sequences with 100% identity to *Penicillium citrinum* and *Simplicillium lamellicola*, with EMIRGE and phyloFlash also producing 18S and mitochondrial 16S sequences with high identity to these species.

Out of the pipelines tested, denim was most successful in identifying the species in the benchmarking dataset. The lower number of sequences generated by EMIRGE and phyloFlash was likely due to the conserved nature of 18S and mitochondrial 16S genes within the genus *Phytophthora*. This also limits the utility of the sequences for taxonomic identification. The inability of denim to identify all species within the dataset, and the fact that only one full ITS sequence could be generated, may point to the difficulty in assembling ITS regions using very short (2x90 bp) reads, particularly within the conserved 5.8S region.

### ITS sequences from genomes and metagenomes

A total of 733 unique full ITS sequences were assembled from the 104 datasets, out of which 544 were assembled from genomic datasets, 181 were assembled from metagenomic datasets and 8 were identically assembled from both genomic and metagenomic datasets. The total number of “complete” (i.e. not on the border of a contig) ITS1/ITS2 region sequences assembled were 1626 and 2191 respectively, out of which 1203 (ITS1) and 1715 (ITS2) were assembled from genomic datasets, 395 (ITS1) and 451 (ITS2) were assembled from metagenomic datasets and 28 (ITS1) and 25 (ITS2) were assembled from both genomic and metagenomic datasets. The assembled sequences are affiliated with a diverse set of eukaryotes, with most originating from *Trachaeophyta*, *Fungi*, *Oomycota* and *Metazoa* (Fig. 2A, Supplementary Table 4). The dominance of *Trachaeophyta* (vascular plants) may reflect the large amounts of plant genome sequencing data within the current dataset.

Variations in sequence length and GC content were present between the different taxonomic groups: *Oomycota* and *Metazoa* ITS sequences were relatively long, while *Trachaeophyta* sequences had relatively high GC contents (Fig. 2B). Moreover, the sequence lengths and GC contents of *Metazoa* ITS sequences were variable in comparison to other lineages (Fig. 2C). Since long sequences and high GC contents are associated with low PCR amplification efficiency [41], these groups may be affected to a greater extent by PCR bias. In addition, amplicon sequencing strategies where forward and reverse reads are merged based on overlap may not be able to capture the full diversity of lineages with longer ITS sequences. When using a 2x250 bp paired-end setup and merging reads based on 50 bp overlaps (as has been performed for *Oomycota* [10]), sequences of up 400 bp (including primers) can be merged. As 6.82% (*Oomycota*) and 36.2% (*Metazoa*) of ITS1 sequences, and 93.2% (*Oomycota*) and 17.9% (*Metazoa*) of ITS2 sequences exceed 400 bp, the full diversity of these lineages may be underrepresented in amplicon sequencing studies. Contrastingly, only 1.47% of ITS1 sequences and 2.13% of ITS2 sequences exceed 400 bp in *Fungi*.

UCHIME analysis identified 25 full ITS sequences, 69 ITS1 sequences and 27 ITS2 sequences as potentially chimeric, representing 3.41%, 4.24% and 1.23% of all sequences in the respective categories (Supplementary Fig. 1). Potentially chimeric sequences were particularly prevalent in *Trachaeophyta* ITS1 sequences, where 5.27% of all sequences were marked. This may be an effect of high degrees of natural biological recombination of ITS sequences within plants [42].

The estimated fraction of chimeric sequences assembled by the denim pipeline exceeds the fraction of potentially chimeric fungal sequences within the UNITE/INSDC databases (1.4%) [43]. As such, caution should be taken when further interpreting the assembled ITS sequences, and further improvements to the denim pipeline should aim to reduce the formation of chimeric sequences.

### Phylogenetic placement of *Oomycota* ITS sequences

The taxonomic identities of the *Oomycota* ITS sequences were diverse: only a minority (13/73 full ITS sequences, 14/102 ITS1 sequences and 7/88 ITS2 sequences) could be conclusively associated to the genus *Phytophthora* by the GBIF sequence ID tool. A higher number of sequences were associated with genus *Pythium* (14/73 full ITS sequences, 17/102 ITS1 sequences and 15/88 ITS2 sequences). The remaining ITS sequences were associated with a broad range of genera including *Albugo*, *Aphanomyces*, *Globisporangium, Phytopythium* and *Pythiogeton*, as well as downy mildew genera such as *Peronospora*, *Hyaloperonospora* and *Plasmopara*.

#### Phytophthora

ITS1, ITS2, and full sequences annotated as *Phytophthora* formed well supported clades in their respective phylogenetic trees (UFboot = 94%, 97% and 99% respectively). Assembled ITS sequences were affiliated with *Phytophthora* clades 1, 2, 4, 6, 7 and 8, as well as downy mildew genera (clades 15 and 16), as defined by Abad et al [5].

*Phytophthora* ITS sequences in clade 1 were affiliated with clade 1a (one full ITS sequence related to *P. aleatoria*, from an *Alternaria panax* genome sequencing project), clade 1c (one full ITS sequence and two ITS1 sequences, all related to *P. infestans*, from *Synchytrium endobitoticum* metagenome sequencing, *Magallana ariakensis* genome sequencing and *Vitis vinifera* metagenome sequencing projects) and clade 1d (one full ITS sequence and two ITS2 sequences, related to *P. nicotianae*, from *Nicotiana tabacum* and *Citrus* spp. metagenome sequencing projects).

*Phytophthora* ITS sequences in clade 2 were affiliated with clade 2a (one full ITS sequence, related to *P. citrophthora* and *P. meadii*, from a soil metagenome sequencing project), clade 2b (two full ITS sequences, four ITS1 sequences and two ITS2 sequences, related to *P. capsici* and *P. aysenensis*, from *Marasmius tenuissimus* genome sequencing, *Amaranthus caudatus* genome sequencing, *Capsicum annuum* metagenome sequencing and *Theobroma cacao* fermentation metagenome sequencing projects) and clade 2e (one full ITS sequence, related to *P. frigida*, from a *Tylosema esculentum* genome sequencing project),

One full ITS sequence, related to *P. megakarya*, was affiliated with clade 4, and was assembled from a *Marasmius tenuissimus* genome sequencing project.

*Phytophthora* ITS sequences in clade 6 were affiliated with clade 6a (one full ITS sequence and one ITS2 sequence, related to *P. rosacearum* and *P.* taxon sylvatica-like, from soil metagenome sequencing and sewage discharge biofilm metagenome sequencing projects), clade 6b (one ITS1 sequence, related to *P. litoralis* and *P. crassamura*, from a sewage discharge biofilm metagenome sequencing project) and clade 6d (one ITS1 sequence, related to *P. lacustris*, from a sewage discharge biofilm metagenome sequencing project).

*Phytophthora* ITS sequences in clade 7 were affiliated with clade 7b (one full ITS sequence and two ITS1 sequences, all related to *P. asiatica*, from *Gossypium darwinii* genome sequencing, *Homo sapiens* stool metagenome sequencing, and *Enterobacter hormaechei* genome sequencing projects) and clade 7c (one full ITS sequence, related to *P. cinnamomi*, assembled from a *H. sapiens* genome sequencing project).

*Phytophthora* ITS sequences in clade 8 were affiliated with clade 8a (one ITS1 sequence, related to *P. medicaginis*, from a *Caenorhabditis elegans* genome sequencing project).

In addition to sequences associated with *Phytophthora* lineages, several sequences affiliated with downy mildew genera, which phylogenetically cluster within *Phytophthora*, were assembled. Five ITS1 sequences and four ITS2 sequences were associated with genus *Plasmopara*, originating from *Euphrasia arctica* and *V. vinifera* genome sequencing projects. Seven ITS1 sequences and nine ITS2 sequences were associated with the genus *Peronospora*, originating from *Antirrhinum majus* genome sequencing, *Myosotis brevis* genome sequencing, *Corydalis* spp. genome sequencing, *Myosurus minimus* genome sequencing, *Fagopyrum esculentum* genome sequencing, air metagenome sequencing projects. Twelve ITS1 sequences and 11 ITS2 sequences were associated with the genus *Hyaloperonospora*, originating from *Arabis alpina* genome sequencing, *Brassica juncea* genome sequencing, *Brassica napus* genome sequencing, *Arabidopsis thaliana* genome sequencing, *Arabidopsis arenosa* genome sequencing, *Arabidopsis lyrata* genome sequencing, *Raphanus sativus* compost metagenome sequencing and air microbiome sequencing projects.

More divergent *Phytophthora* sequences were also assembled. One ITS1 sequence from an *Oryza sativa* genome sequencing project clustered with *P. pseudorosacearum* but did not match closely to sequences in the dataset or any sequences within the NCBI non-redundant (nr) database, as the closest hit (“Uncultured *Plasmopara* clone OTU0510”, accession MN269295.1) had only 86.7% identity over 30% of the sequence. Similarly, one ITS1 sequence from a *Myositis brevis* genome sequencing projects had an uncertain phylogenetic placement and only 90.8% identity (100% coverage) to the closest hit in the nr database (“*Peronospora* sp. voucher GLM-F68067”, accession PP528459.1). Additionally, one ITS2 from an *A. arenosa* sequencing project, which clustered with *Peronospora erophilae*, had only 86.8% identity (100% coverage) to the closest hit in the nr database (“*Hyaloperonospora thlaspeos-perfoliati* strain MG 17-9”, accession AY531432.1).

The diverse set of *Phytophthora* ITS sequences assembled in the current study indicates the utility of the denim approach both as a method of discovering and tracking potential hosts and vectors of pathogenic eukaryotes, and as a method of discovering novel lineages. The geographic information associated with the analyzed studies can serve as a global complement to existent monitoring efforts, and source organism metadata from genomic sequencing studies may point to hitherto unknown plant populations vulnerable to *Phytophthora* or currently unregulated routes through which invasive *Phytophthora* strains can spread. For example, while *P. frigida* has been reported to infect and damage other members of family *Fabaceae*, such as *Akacia mearnsii* [44], the current study assembled a full-length ITS sequence closely related to *P. frigida* from a *T. esculentum* genome sequencing project. *T. esculentum*, which is being bred into a commercial drought-resistant leguminous crop [45], may thus be vulnerable to infection by *P. frigida*, potentially hampering domestication efforts. Future studies could serve to verify this potential host-pathogen interaction through pathogenicity trials.

The presence of *Phytophthora*-associated ITS sequences within non-host genome sequencing projects highlights the existence of cryptic ecological reservoirs. These sequences could represent dormant propagules, transient colonisation, or incidental environmental uptake, emphasizing that *Phytophthora* occupies a broader ecological space than the traditional plant–soil paradigm. Such overlooked reservoirs may play a role in pathogen persistence and dispersal at landscape scales.

#### Other *Oomycota*

Aside from *Phytophthora*, *Oomycota* ITS sequences associated with other genera were assembled: *Achlya* (one full ITS sequence, assembled from a soil metagenome sequencing project), *Aphanomyces* (three full ITS sequences, assembled from an *Anopheles gambiae* genome sequencing project and a *Synchytrium endobioticum* sequencing project), *Globisporangium* (five full ITS sequences, four ITS1 sequences and four ITS2 sequences, from plastisphere metagenome sequencing, sewage discharge biofilm metagenome sequencing, *Bidens pilosa* genome sequencing, *B. napus* genome sequencing, *Diabrotica virgifera* genome sequencing, *Podila minutissima* genome sequencing, *Prunus mira* genome sequencing projects), *Phytopythium* (two full ITS sequences, two ITS1 sequences and one ITS2 sequence, assembled from sewage discharge biofilm metagenome sequencing, *B. pilosa* genome sequencing and *Orobanche cernua* genome sequencing projects), *Pythiogeton* (one full ITS sequence, five ITS1 sequences and two ITS2 sequences, assembled from plastisphere metagenome sequencing and soil metagenome sequencing projects), *Pythium* (14 full ITS sequences, six ITS1 sequences and four ITS2 sequences, assembled from *N. tabacum* metagenome sequencing, sewage discharge biofilm metagenome sequencing, soil metagenome sequencing, *A. caudatus* genome sequencing, *A. gambiae* genome sequencing, *B. pilosa* genome sequencing, *Brachypodium distachyon* genome sequencing, *Daphnia magna* genome sequencing, *Gasterosteus aculeatus* genome sequencing, *Lemna minuta* genome sequencing and *Mortierella* sp. AD030 genome sequencing projects) and *Saprolegnia* (one full ITS sequence, two ITS1 sequences and one ITS2 sequence, from sewage discharge biofilm metagenome sequencing and *G. aculeatus* genome sequencing projects).

A potentially concerning findings is the assembly of a full ITS sequence with 99.8% identity to that of known human pathogen *Pythium insidiosum* from an African mosquito (*A. gambiae*) genome sequencing project [46]. *P. insidisoum* is the causative agent of pythiosis, a life-threatening infection which is emerging in Africa [47]. While it should be noted that the presence of DNA marker genes do not provide evidence of infectivity or viability, *Pythium insidiosum* has previously been shown to infect other mosquito species (*Aedes aegypti*) in Brazil [48].

#### Limitations of the denim approach

While the approach utilized in this manuscript has the potential to identify undetected *Phytophthora* lineages and other taxa of interest, several steps involved limit it from being a truly comprehensive search. The process of identifying suitable datasets with high *Phytophthora* abundance is an initial limitation, as the reliance on STAT annotations requires the lineages present to have significant genetic overlap with species in the RefSeq genomes collection. Updating the STAT annotations to cover more species would likely improve the recovery of more divergent lineages. For example, expanding the reference database to include all *Phytophthora* reference genomes present in the NCBI genome portal would increase the number of species represented from 4 to 80 (queried 2025-11-10).

The denim pipeline also presents some limitations. The insertion of introns longer than the read length into the ITS region would cause reads originating from these introns to be removed in the mapping step, preventing ITS assembly unless the intron sequence has >50% identity to reference sequences within the utilized database. Such insertions have been observed in obligately biotrophic *Phytophthora* species such as *P. cyperi*, where the ITS1 sequence contains a 1300 bp intron [49], and *Bremia lactucae*, where the ITS2 sequence contains a 1683 bp intron [50]. The presence of repeat arrays longer than the read length would additionally limit denim’s ability to accurately assemble ITS sequences. Rearrangements and deletions within the rRNA operon may prevent ITSx from identifying ITS sequences and may also limit their utility as informative markers. Future pipelines for assembly of ITS sequences would thus benefit from longer read lengths and may additionally benefit from filtering reads based on identity to more conservative markers (such as 18S, 5.8S and 28S regions) prior to assembly of more informative markers (ITS1 and ITS2).

As with many DNA-based analysis methods, the method described in this manuscript is incapable of discerning species from interspecific hybrids in a metagenome, as it is not possible to tell when two ITS sequences originate from the same cell. Proximity-ligation methods, where closely situated DNA molecules are joined together prior to sequencing [51], could make this possible in the future.

## Conclusions

This study presents a methodological advance in our abilities to detect and identify eukaryotes in shotgun sequencing data. Assembling ITS sequences from genomes and metagenomes provides a way of increasing our understanding of the host preference, geographic distribution, and phylogenetic diversity of pathogenic eukaryotes, such as *Phytophthora*. The utility of the approach is not restricted to pathogens but could be used for any lineage for which “invisible” microbes are suspected to exist. With appropriate quality controls, the resulting sequences could be used to expand or complement existing reference databases. Utilizing these expanded reference databases with denim in an iterative process may allow discovery of lineages even further removed from those present in reference databases. If assembled sequences are used in this manner, we strongly suggest that they are labelled as putative until it has been confirmed that they are not chimeric or pseudogenes. Marker gene sequences could be considered non-chimeric if a substantial number of reads covering the entire sequence are observed with 100% identity to the assembled sequence. Sequences could be considered non-pseudogenic if they are observed as the only identified ITS sequence within a genome or a near-complete metagenome-assembled genome.

Extending the denim pipeline to support analysis of long reads, such as those produced by sequencing platforms developed by Oxford Nanopore or Pacific Biosciences, may reduce chimera formation and improve the recovery of ITS sequences with low identity to those available in reference databases. As the ITS1, ITS2 and 5.8S rRNA regions can all be contained on a single long read, chimeric ITS sequences are less likely to be formed, improving the trustworthiness of the results. The reliance of the pipeline on reference databases could also be reduced by screening long reads for rRNA coding genes using hidden Markov models and assembling the matching reads. In contrast to the current approach (mapping short reads to ITS sequence databases), this would only require the rRNA genes to be conserved, permitting assembly of flanking ITS sequences which do not match any database.

While the methodological advances presented in this study have been framed with regards to their utility for studying the genus *Phytophthora*, they are equally applicable for other eukaryotic organisms. As such, denim, particularly when paired with taxonomic screening of large sequencing databases, serves as a new tool for passive global ecological surveillance, which can reveal unexpected microbial niches and host associations. It could potentially help uncover cryptic biodiversity, and identify unknown habitats and potential introduction pathways, to aid in preventative measures or surveillance efforts.

## Supplementary Material

Supplementary_information_resubmission_2_ycag019

## Data Availability

Genomic sequencing and metagenomic sequencing data are available from the Sequence Read Archive; all accessions used in this study are listed in Supplementary Table 1. Assembled ITS sequences (https://figshare.com/s/6c5cee3073ac3e7f65ea) and generated phylogenetic trees (https://figshare.com/s/26cf7fe440a0a52aa607) are available through figshare.
