# Supplementary material for: Detecting “invisible” Phytophthora lineages in publicly available sequencing data
Source: ISME Commun. 2026 Jan 30;6(1):ycag019. doi: 10.1093/ismeco/ycag019 (PMC12915580; doi:10.1093/ismeco/ycag019)
Supplement: Supplementary_information_resubmission_2_ycag019 [file supplementary_information_resubmission_2_ycag019.docx]

Supplementary information for *Detecting “invisible” Phytophthora lineages in publicly available sequencing data*

Tage Rosenqvist^1^*, Michelle Cleary^1^

1 Swedish University of Agricultural Sciences, Southern Swedish Forest Research Centre, Sundsvägen 3, 230 53 Alnarp, Sweden

* corresponding author

# Supplementary tables

Supplementary table 1: Sequencing datasets used for benchmarking in this study.

| Accession ID | Species |
| --- | --- |
| SRR16202028 | *Phytophthora brassicae* |
| SRR16202022 | *Phytophthora foliorum* |
| SRR16202018 | *Phytophthora hibernalis* |
| SRR16202020 | *Phytophthora melonis* |
| SRR16202019 | *Phytophthora niederhauserii* |
| SRR16202018 | *Phytophthora parvispora* |
| SRR16202023 | *Phytophthora pini* |
| SRR16202017 | *Phytophthora pisi* |
| SRR16202016 | *Phytophthora pistaciae* |
| SRR16202025 | *Phytophthora syringiae* |

Supplementary table 2: Sequencing datasets analyzed in this study.

| Accession ID | Type | Organism/environment | Bases (Gbp) |
| --- | --- | --- | --- |
| DRR438133 | Genomic | *Fagopyrum esculentum* | 20.8 |
| ERR10738536 | Genomic | *Oryza sativa* | 5.8 |
| ERR12736058 | Genomic | *Arabidopsis lyrata* | 11.8 |
| ERR13245338 | Genomic | *Halopteris paniculata* | 27.6 |
| ERR2124224 | Genomic | *Antirrhinum majus* | 22.5 |
| ERR267883 | Genomic | *Solanum tuberosum* | 6.8 |
| ERR3021440 | Genomic | *Amaranthus caudatus* | 4.6 |
| ERR3148763 | Genomic | *Solanum tuberosum* | 0.244 |
| ERR5100683 | Genomic | *Arabidopsis thaliana* | 3.2 |
| ERR5529542 | Genomic | *Myosurus minimus* | 0.967 |
| ERR6590515 | Genomic | *Gossypium darwinii* | 11.2 |
| ERR7256390 | Genomic | *Mustela putorius* | 30.2 |
| ERR7412459 | Genomic | *Ambrosia artemisiifolia* | 34.4 |
| ERR9629360 | Genomic | *Anopheles gambiae* | 12.0 |
| ERR979157 | Genomic | *Equus ferus* | 2.1 |
| SRR10416192 | Genomic | *Broussonetia papyrifera* | 0.466 |
| SRR10549958 | Genomic | *Arabidopsis arenosa* | 10.9 |
| SRR10694816 | Genomic | *Homo sapiens* | 12.1 |
| SRR1106544 | Genomic | *Diabrotica virgifera* | 29.1 |
| SRR11164847 | Genomic | *Saccharomyces cerevisiae* | 2.6 |
| SRR11183106 | Genomic | *Acipenser schrenckii* | 40.3 |
| SRR11906258 | Genomic | *Anas platyrhynchos* | 8.6 |
| SRR11963570 | Genomic | *Brassica juncea* | 17.4 |
| SRR12420117 | Genomic | *Vigna radiata* | 2.6 |
| SRR12628148 | Genomic | *Zea mays* | 3.4 |
| SRR13154262 | Genomic | *Drosophila melanogaster* | 36.8 |
| SRR13567785 | Genomic | *Glycine max* | 6.0 |
| SRR14278830 | Genomic | *Brassica juncea* | 14.0 |
| SRR14864938 | Genomic | *Magallana ariakensis* | 13.0 |
| SRR15692328 | Genomic | *Brassica napus* | 37.9 |
| SRR16382375 | Genomic | *Homo sapiens* | 65.9 |
| SRR16538696 | Genomic | *Arabis alpina* | 8.2 |
| SRR16873150 | Genomic | *Allium cepa* | 4.0 |
| SRR17267512 | Genomic | *Orobanche cernua* | 29.6 |
| SRR17685681 | Genomic | *Brassica oleracea* | 7.3 |
| SRR18051247 | Genomic | *Brassica napus* | 17.6 |
| SRR18888509 | Genomic | *Euphrasia arctica* | 45.1 |
| SRR1980958 | Genomic | *Stylophora pistillata* | 30.4 |
| SRR21541472 | Genomic | *Tylosema esculentum* | 18.6 |
| SRR21869639 | Genomic | *Anas platyrhynchos* | 7.8 |
| SRR21902265 | Genomic | *Caenorhabditis elegans* | 3.1 |
| SRR22198005 | Genomic | *Adenophora remotiflora* | 5.4 |
| SRR22243581 | Genomic | *Bidens pilosa* | 43.5 |
| SRR2392052 | Genomic | *Solanum lycopersicum x Solanum pimpinellifolium* | 1.2 |
| SRR24289006 | Genomic | *Oryza sativa* | 29.9 |
| SRR26387456 | Genomic | *Vitis vinifera* | 57.7 |
| SRR26391463 | Genomic | *Vitis pseudoreticulata* | 30.4 |
| SRR26394877 | Genomic | *Daphnia magna* | 18.3 |
| SRR27398444 | Genomic | *Kwoniella pini* | 9.1 |
| SRR27830168 | Genomic | *Acer griseum* | 0.730 |
| SRR27840079 | Genomic | *Lemna minuta* | 20.9 |
| SRR27991391 | Genomic | *Marasmius tenuissimus* | 11.3 |
| SRR27997387 | Genomic | *Brassica napus* | 38.0 |
| SRR2824356 | Genomic | *Oryza sativa* | 29.4 |
| SRR28289130 | Genomic | *Corydalis sp.* | 8.9 |
| SRR29484333 | Genomic | *Myositis brevis* | 1.4 |
| SRR30830351 | Genomic | *Homo sapiens* | 32.2 |
| SRR3237755 | Genomic | *Prunus mira* | 12.2 |
| SRR32793405 | Genomic | *Odontolabis cuvera* | 48.7 |
| SRR3386339 | Genomic | *Alternaria panax* | 14.7 |
| SRR350458 | Genomic | *Tetrahymena thermophila* | 4.3 |
| SRR5190897 | Genomic | *Mortierella sp. AD030* | 0.556 |
| SRR5192046 | Genomic | *Mortierella minutissima* | 0.631 |
| SRR5421752 | Genomic | *Arabidopsis thaliana* | 10.9 |
| SRR5837540 | Genomic | *Citrus reticulata* | 5.1 |
| SRR5891919 | Genomic | *Vitis girdiana* | 8.1 |
| SRR6111443 | Genomic | *Bombyx mori* | 6.8 |
| SRR6838397 | Genomic | *Gasterosteus aculeatus* | 27.8 |
| SRR7630991 | Genomic | *Brassica napus* | 10.5 |
| SRR7716625 | Genomic | *Enterobacter hormaechei* | 1.9 |
| SRR7875443 | Genomic | *Bos grunniens* | 32.8 |
| SRR7880915 | Genomic | *Arabis alpina* | 4.9 |
| SRR8143226 | Genomic | *Solanum tuberosum* | 16.9 |
| SRR8372088 | Genomic | *Brachypodium distachyon* | 13.3 |
| SRR8377371 | Genomic | *Oryza sativa* | 5.4 |
| SRR8427075 | Genomic | *Cryptococcus deuterogattii* | 6.1 |
| SRR8586074 | Genomic | *Malus domestica* | 28.5 |
| SRR8727822 | Genomic | *Vitis vinifera* | 15.1 |
| SRR8835160 | Genomic | *Vitis vinifera* | 7.9 |
| SRR8935388 | Genomic | *Phaseolus vulgaris* | 1.7 |
| DRR249176 | Metagenomic | Human blood | 0.949 |
| ERR10641114 | Metagenomic | *Synchytrium endobioticum* resting spores | 6.9 |
| ERR13725843 | Metagenomic | *Vitis vinifera* leaves | 12.4 |
| ERR14218477 | Metagenomic | *Vitis vinifera* leaves | 1.2 |
| ERR3192248 | Metagenomic | *Arabidopsis thaliana* rosettes | 23.1 |
| SRR11602142 | Metagenomic | *Theobroma cacao* bean fermentation | 8.5 |
| SRR11835836 | Metagenomic | Human stool | 8.9 |
| SRR12778019 | Metagenomic | *Capsicum annuum* | 27.4 |
| SRR14117416 | Metagenomic | Soil | 11.6 |
| SRR14301316 | Metagenomic | *Raphanus sativus* peel compost | 12.6 |
| SRR17300690 | Metagenomic | Air | 1.3 |
| SRR17656794 | Metagenomic | Plastisphere | 21.1 |
| SRR17972516 | Metagenomic | *Erysiphe necator* lesions in *Vitis vinifera* | 12.2 |
| SRR20818738 | Metagenomic | Anaerobic sludge | 6.7 |
| SRR22222713 | Metagenomic | Human gut | 4.0 |
| SRR25158288 | Metagenomic | Soil | 33.4 |
| SRR25485500 | Metagenomic | *Nicotiana tabacum* | 16.1 |
| SRR27290611 | Metagenomic | *Amorphophallus muelleri* | 12.5 |
| SRR28145606 | Metagenomic | Soil | 11.6 |
| SRR30970440 | Metagenomic | Soil | 22.8 |
| SRR32051688 | Metagenomic | Sewage discharge basin biofilm | 30.1 |
| SRR32058888 | Metagenomic | *Nicotiana tabacum* roots | 7.4 |
| SRR5195108 | Metagenomic | *Citrus* rhizosphere | 34.4 |
| SRR7842619 | Metagenomic | Partial nitritation-anammox reactor | 24.0 |

Supplementary table 3: BLASTn results of marker gene sequences from the benchmarking assay. In cases of multiple, identically similar BLASTn hits, results labelled as ex-type/ex-epitype are listed. If none of the multiple hits are listed as ex-types/ex-epitypes, the first hit is listed. Bolded species names were part of the benchmarking dataset. *ex-type/ex-epitype. **also matched 100% with P. pini.

| Sequence name | Software | Marker | Closest hit | Identity (%) |
| --- | --- | --- | --- | --- |
| k141_18\|O\|ITS1 Extracted ITS1 sequence 128-312 (185 bp) | denim | ITS1 | ***Phytophthora pini**** (MG865565.1) | 100 |
| k141_75\|O\|ITS1 Extracted ITS1 sequence 236-468 (233 bp) | denim | ITS1 | *Phytophthora sojae* (GU259178.1) | 99.6 |
| k141_3\|O\|ITS1 Extracted ITS1 sequence 105-306 (202 bp) | denim | ITS1 | ***Phytophthora brassicae**** (MG783384.1) | 100 |
| k141_214\|F\|ITS1 Extracted ITS1 sequence 2267-2405 (139 bp) | denim | ITS1 | *Penicillium citrinum* (PX207720.1) | 100 |
| k141_215\|F\|ITS1 Extracted ITS1 sequence 2249-2429 (181 bp) | denim | ITS1 | *Verticillium* sp. F-1406 WF (DQ211519.1) | 100 |
| k141_114\|O\|ITS1 Extracted ITS1 sequence 55-287 (233 bp) | denim | ITS1 | ***Phytophthora pistaciae*** (FJ746648.1) | 100 |
| k141_171\|O\|ITS1 Extracted ITS1 sequence 437-643 (207 bp) | denim | ITS1 | ***Phytophthora syringae*** (HQ643361.1) | 100 |
| k141_103\|O\|ITS1 Extracted ITS1 sequence 56-576 (521 bp) | denim | ITS1 | ***Phytophthora melonis*** (OK606119.1) | 99.4 |
| k141_69\|O\|ITS1 Extracted ITS1 sequence 105-307 (203 bp) | denim | ITS1 | ***Phytophthora foliorum**** (MG865492.1) | 100 |
| k141_14\|O\|ITS2 Extracted ITS2 sequence 174-618 (445 bp) | denim | ITS2 | ***Phytophthora brassicae**** (MG783384.1) | 99.6 |
| k141_214\|F\|ITS2 Extracted ITS2 sequence 2564-2729 (166 bp) | denim | ITS2 | *Penicillium citrinum* (MT560285.1) | 100 |
| k141_215\|F\|ITS2 Extracted ITS2 sequence 2588-2765 (178 bp) | denim | ITS2 | *Simplicillium lamellicola* (MH859670.1) | 100 |
| k141_122\|O\|ITS2 Extracted ITS2 sequence 86-518 (433 bp) | denim | ITS2 | ***Phytophthora pistaciae*** (AF403505.1) | 99.5 |
| k141_207\|O\|ITS2 Extracted ITS2 sequence 96-1131 (1036 bp) | denim | ITS2 | ***Phytophthora cinnamomi var. parvispora*** (HQ643200.1) | 99.2 |
| k141_164\|O\|ITS2 Extracted ITS2 sequence 127-572 (446 bp) | denim | ITS2 | ***Phytophthora syringae*** (MH750851.1) | 100 |
| k141_171\|O\|ITS2 Extracted ITS2 sequence 803-1218 (416 bp) | denim | ITS2 | *Phytophthora citricola*** (MF980709.1) | 100 |
| pf_out.PFemirge_1280_0.555735 | EMIRGE | 18S | *Phytophthora megasperma* (X54265.1) | 99.8 |
| pf_out.PFemirge_2194_0.199776 | EMIRGE | Mito. 16S | *Phytophthora sojae* (NC_009385.1) | 99.5 |
| pf_out.PFemirge_2273_0.123148 | EMIRGE | 18S | *Phytophthora sojae* (JN635166.1) | 99.9 |
| pf_out.PFemirge_2683_0.036673 | EMIRGE | Mito. 16S | *Phytophthora cinnamomi* (PP108238.1) | 96.7 |
| pf_out.PFemirge_2529_0.051319 | EMIRGE | 18S | *Phytophthora infestans* T30-4 (XR_009934735.1) | 98.8 |
| pf_out.PFemirge_2684_0.013738 | EMIRGE | Mito. 16S | *Phytophthora citricola* (NC_067070.1) | 94.9 |
| pf_out.PFemirge_652_0.006305 | EMIRGE | 18S | *Penicillium* sp. strain JMET 19 (MZ892607.1) | 99.4 |
| pf_out.PFemirge_27_0.003925 | EMIRGE | 18S | *Protoblastenia lilacina* (AY548825.1) | 90.4 |
| pf_out.PFemirge_2203_0.003742 | EMIRGE | Mito. 16S | *Simplicillium lamellicola* (OZ124174.1) | 99.0 |
| pf_out.PFemirge_1546_0.002758 | EMIRGE | 18S | *Fungal* sp. FCAS129 (GQ120159.1) | 96.5 |
| pf_out.PFemirge_126_0.001420 | EMIRGE | 18S | Uncultured *Dikarya* clone AY2009B1 (HQ219403.1) | 97.4 |
| pf_out.PFemirge_2258_0.001421 | EMIRGE | Mito. 16S | *Talaromyces apiculatus* (OR752424.1) | 99.6 |
| pf_out.PFemirge_2540_0.000040 | EMIRGE | 18S | *Penicillium citrinum* (MH990629.1) | 99.7 |
| pf_out.PFspades_1_525.248770 | phyloFlash | Mito. 16S | *Phytophthora sojae* (NC_009385.1) | 99.6 |
| pf_out.PFspades_2_5.388743 | phyloFlash | Mito. 16S | *Penicillium* sp. strain D1806 (MN960691.1) | 99.9 |
| pf_out.PFspades_4_23.960562 | phyloFlash | 18S | *Penicillium citrinum* (MH990629.1) | 100 |
| pf_out.PFspades_5_9.983106 | phyloFlash | 18S | Uncultured *Dikarya* clone AY2009A16 (HQ219397.1) | 100 |

Supplementary table 4: Number of unique ITS sequences generated by denim, per eukaryotic lineage (as determined by ITSx).

| Lineage | Full ITS | ITS1 | ITS2 |
| --- | --- | --- | --- |
| *Alveolata* | 18 | 37 | 10 |
| *Amoebozoa* | 2 | 0 | 0 |
| *Bacillariophyta* | 0 | 2 | 1 |
| *Bryophyta* | 1 | 4 | 2 |
| *Chlorophyta* | 6 | 32 | 11 |
| *Euglenozoa* | 2 | 8 | 4 |
| *Eustigmatophyceae* | 0 | 1 | 0 |
| *Fungi* | 154 | 272 | 282 |
| *Metazoa* | 45 | 69 | 67 |
| *Oomycota* | 73 | 102 | 88 |
| *Phaeophyceae* | 3 | 16 | 9 |
| *Rhizaria* | 7 | 16 | 6 |
| *Rhodophyta* | 0 | 3 | 1 |
| *Synoruphyceae* | 2 | 2 | 3 |
| *Trachaeophyta* | 420 | 1062 | 1707 |

# Supplementary figures


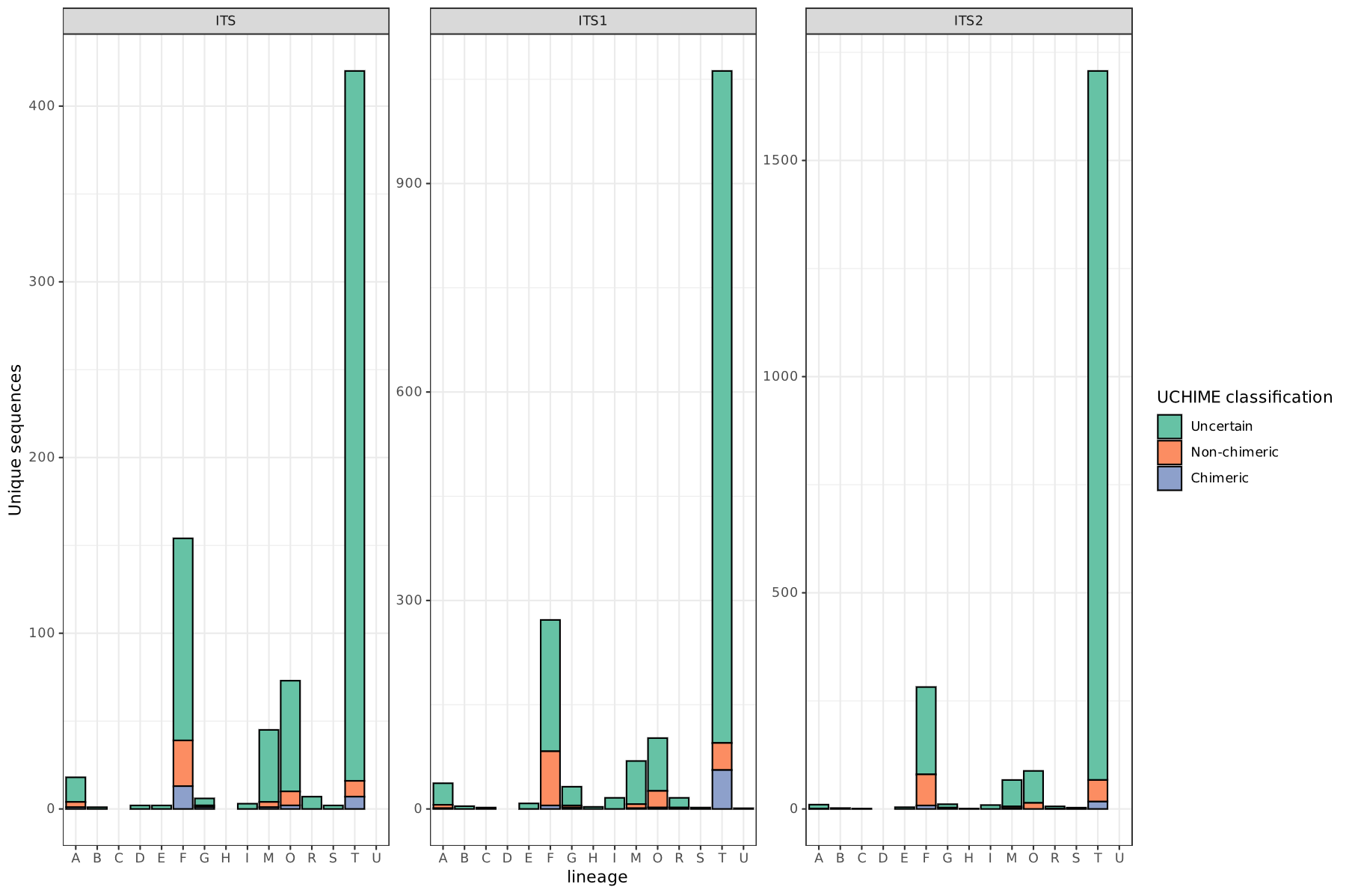


Supplementary figure 1: UCHIME classifications for assembled sequences.
